# Skilled Care at Birth among Rural Women in Nepal: Practice and Challenges

**DOI:** 10.3329/jhpn.v29i4.8453

**Published:** 2011-08

**Authors:** Sulochana Dhakal, Edwin van Teijlingen, Edwin Amalraj Raja, Keshar Bahadur Dhakal

**Affiliations:** ^1^Section of Population Health, University of Aberdeen, Aberdeen, UK; ^2^School of Health and Social Care, Bournemouth University, Bournemouth, UK,; ^3^Mid-western Regional Hospital, Birendranagar, Surkhet, Nepal

**Keywords:** Childbirth, Cross-sectional studies, Descriptive studies, Delivery, Rural health services, Skilled attendants, Nepal

## Abstract

In Nepal, most births take place at home, and many, particularly in rural areas, are not attended by a skilled birth attendant. The main objectives of the study were to assess the use of skilled delivery care and barriers to access such care in a rural community and to assess health problems during delivery and seeking care. This cross-sectional study was carried out in two Village Development Committees in Nepal in 2006. In total, 150 women who had a live birth in the 24 months preceding the survey were interviewed using a structured questionnaire. The sample population included married women aged 15-49 years. Forty-six (31%) women delivered their babies at hospital, and 104 (69%) delivered at home. The cost of delivery at hospital was significantly (p<0.001) higher than that of a delivery at home. Results of univariate analysis showed that women from Brahmin-Chhetri ethnicity, women with higher education or who were more skilled, whose husbands had higher education and more skilled jobs, had first or second childbirth, and having adverse previous obstetric history were associated with institutional delivery while women with higher education and having an adverse history of pregnancy outcome predicted the uptake of skilled delivery care in Nepal. The main perceived problems to access skilled delivery care were: distance to hospital, lack of transportation, lack of awareness on delivery care, and cost. The main reasons for seeking intrapartum care were long labour, retained placenta, and excessive bleeding. Only a quarter of women sought care immediately after problems occurred. The main reasons seeking care late were: the woman or her family not perceiving that there was a serious problem, distance to health facility, and lack of transport. The use of skilled birth attendants at delivery among rural women in Nepal is very poor. Home delivery by unskilled birth attendants is still a common practice among them. Many associated factors relating to the use of skilled delivery care that were identified included age, education and occupation of women, and education and occupation of husbands. Therefore, the availability of skilled delivery care services at the community, initiation of a primary health centre with skilled staff for delivery, and increasing awareness among women to seek skilled delivery care are the best solution.

## INTRODUCTION

In Nepal, most (81%) deliveries take place at home (1). Traditional birth attendants (TBAs) and unskilled birth attendants, such as family members and relatives, are common while some women (7%) give birth without any support (1). Evidence suggests that having skilled attendants at delivery is one of the key interventions for reducing maternal mortality (2). Developing countries where professional attendants are used at delivery have reduced maternal mortality up to 50 per 100,000 livebirths (3). In Nepal, the main barrier to delivery care is the shortage of skilled birth attendants (SBAs), particularly in most rural areas. Local TBAs are not recognized as SBAs by the World Health Organization (WHO) because they are generally not trained to deal with birth-related complications (4). On the other hand, qualified nurses and doctors are often not available in rural Nepal. Maternal and child health workers (MCHWs), who have a 15-week course on maternal and child health and a further six-week ‘refresher’ course in midwifery skills, are playing a key role as community-level skilled attendants in Nepal (5).

Apart from lack of facilities and staff, poverty often restricts the uptake of skilled delivery care (6). The position of women in the family and level of their decision-making power are also predictors of the place of birth. Women with more decision-making power in a household are more than twice as likely to deliver at a health facility compared to those with little power (7). Women who did not have secondary school-level education or who did not earn money and hence did not contribute to the family income were less likely to seek delivery in a healthcare institution in Nepal (8). It is not very clear what characteristics are associated with women seeking institutional delivery. The objectives of this study were, therefore, to assess the use of skilled delivery care and barriers to accessing skilled delivery care in a rural community.

## MATERIALS AND METHODS

### Study design

A descriptive and cross-sectional study was the chosen method to assess immediate information on practice of and barriers to skilled delivery care among respondents at a point in time. It is the best possible study design within limited resources and worsening security situation at the time in Nepal (9). The study was conducted in two Village Development Committees (VDCs): VDC D and C near the Kathmandu valley in Nepal in 2006. The area had a small number of MCHWs and a small community hospital which does not offer emergency obstetric care.

### Participants

The inclusion criteria were ever-married women of reproductive age (15-49 years) who had delivered their last child within 24 months preceding the study and residing in the study areas. If a woman had more than one child aged less than 24 months, only the most recent delivery was considered in the study. Unmarried women, women of beyond reproductive age, and pregnant women were excluded from the study. It was estimated on the basis of latest census data that there were 362 eligible women in the nine wards of the two villages (10). The number of women in our target group may have been overestimated slightly in the census as suggested by Simkhada and colleagues (11). The study subjects were recruited from six of the nine wards in the two villages. Local female community health volunteers (FCHVs) helped provide a sampling frame for each village, from which a random sample of 150 (41% of the target population) women was drawn. Details on selection of eligible women have been earlier reported (12). Visits to houses were made with the help of local FCHVs, with repeat visits if eligible women were not at home. If a selected woman could not be contacted at home after a repeat visit, an eligible woman living next door was included in the study.

### Data-collection

Data were collected using a structured questionnaire, which included both multiple-choice and open-ended questions. A validated questionnaire was adapted from the Nepal Demographic and Health Survey (NDHS) (2001) (13) and Nepal Multiple Indicator Surveillance (NMIS) (1997) fifth cycle (14). Some questions were slightly modified to suit the objectives of the present study. The instrument was revised and improved according to the advice and suggestions made by experts in maternal health.

A pilot study was carried out for pretesting the questionnaire. The pilot study was conducted with eight married women from the study area before main data-collection (15). Women were interviewed in their home using the structured questionnaire. A female qualified nurse (first author) conducted the interviews, and the interviews lasted, on average, for 20 minutes. A copy of the questionnaire is available on request from the first author.

Occupation of woman was categorized as: housewife, farmer, and others (e.g. tailor, business, and teacher) whereas occupation of husbands was categorized as farmer; formal sector and abroad (e.g. civil servant/teacher); and others (e.g. casual labourers, student, driver). Education was categorized as illiterate, primary, and secondary. If a woman had a miscarriage, abortion, or stillbirth in a previous pregnancy, this was categorized as adverse history of pregnancy outcome.

### Analysis of data

Data were analyzed using the SPSS software for Windows (version 13.0). The chi-square test for trend was used for association between ordinal categorical factors, such as education, number of family members, number of children, ethnicity, adverse pregnancy outcomes, and transportation and institutional delivery. The Fisher's exact test was used whenever more than 20% of the cells had the expected value of less than 5, and a continuity correction test was used for finding the associations between binary categories of different perceived barrier factors and institutional delivery care. The odds ratio (OR) and its 95% confidence interval (CI) were calculated to measure the strength of the association among socioeconomic and demographic factors, antenatal care, previous delivery bad outcomes, and institutional delivery. Those factors that were significant at 20% level in univariate analysis were considered for multivariate analysis. Multivariate logistic regression with backward elimination method was used for finding best combination of factors predicting institutional delivery care. A p value of less than 0.05 was considered significant.

### Ethical issues

Ethical approval was obtained from the Nepal Research Council and local authorities. Informed consent was taken from all the respondents, who were assured of anonymity and confidentiality as no personal identifiers were recorded. Moreover, they were assured of their ability to withdraw from the study at any time.

## RESULTS

### Use of skilled delivery care

One hundred and fifty women who had delivered a baby in the previous 24 months were included. Their median age was 23 [interquartile range [(IQR) 21-27] years, with a minimum of 18 years and a maximum of 48 years. More than three-fourths of the women belonged to the Tamang community. Nearly all (96%) were either farmers or housewives, half of the women were illiterate, and half of their husbands were employed.

Forty-six women (31%) had delivered their last baby in hospital while 104 (69%) had delivered their baby at home. Of the deliveries, 8% were assisted by doctors, 23% by nurses, 53% by a family member (mainly mother-in-law), and 7% by a TBA. However, 9% of the women did not get help from anyone at birth, and no single delivery in the home was attended by a skilled attendant.

Only 11 (11%) women had used home delivery-kits (HDK) when they delivered babies at home. Two-thirds of the women who delivered babies at home had used a new or clean blade to cut the cord while one-third had used unclean instruments, such as a sickle or scissor. Half of the women stated that they themselves decided about the place of delivery, 30% said that their mothers-in-law decided for them, and 19% said that their husbands made the decision. As expected, the cost (median, IQR) of delivery at hospital was significantly higher than the cost of delivery at home [Nepalese Rs 2,100 (1,574, 4,000) vs 0 (0, 82.50) (p<0.001)].

### Factors associated with use of delivery care

Results of univariate analysis showed that factors significantly associated with the preference of institutional delivery were women who: (a) achieved secondary school or above education (OR=15.7, 95% CI 6.3-43.8); (b) were housewives (OR=4.8, 95% CI 2.2-1.5) compared to farm workers; (b) had husband with secondary school or above education (OR=3.6, 95% CI 1.1-11.8); (c) had husband working in formal sector or abroad (OR=4.5, 95% CI 1.5-14.1); (d) had received antenatal check-ups (OR=20.0, 95% CI 2.64-151.51); and (e) had experienced adverse pregnancy outcome, (OR=4.2, 95% CI 1.3-13.54). Women were likely to prefer an institutional delivery for their first child (OR=3.92, 95% CI 1.36-11.31) and second child (OR=3.20, 95% CI 1.02-10.01) compared to the third or higher-order pregnancy. Tamang women were less likely to go for institutional delivery than Brahmin/Chhetri ethnic women (OR=0.04, 95% CI 0.01-0.13). Institutional delivery was significantly higher among women residing in VDC D (n=66) than in VDC C [67.4% (n=66) vs 32.6% (n=84), p<0.001].

### Multivariate analysis

Those variables which were found to be significant in univariate analysis were included in multivariate analysis, including area of residence (=VDC), for adjustment. Since there was a strong association between education and occupation, to overcome the multicollinearity problem, the education levels of women and husbands were included. Women who had received secondary education and above (OR=5.61, 95% CI 1.53-20.54) or had a previous history of adverse pregnancy outcomes (OR=9.8, 95% CI 2.31-41.11) were significantly more likely to have sought institutional delivery (Table 1).

### Perceived problems in accessing skilled delivery and suggestions for improvement

Most perceived barriers to access delivery care—(a) health facility is located far away–30.4% vs 42.3; (b) no skilled female health worker at village–9.6% vs 11.5%; (c) woman and family members not aware of delivery care–54.3% vs 43.3%; (d) no delivery care at local level–6.5% vs 3.8%; (e) no time due to work in the home–0% vs 2.9%; and (f) no money–21.7% vs 14.4%—were not significantly different between women delivering their babies at hospital or at home (Table 2). However, Table 1 suggests that more women who delivered at home perceived that lack of transport was a problem relating to accessing hospital (OR=3.3, 95% CI 1.20-9.22).

### Suggestions for improvement of delivery care

Suggestions for the improvement of delivery care in the community were sought from all the women. They recommended that a health facility with delivery care should be available in the village (47%), should increase awareness on delivery care among women/family (25%), should have more medicines (17%) and more family support (17%), and ensure the availability of an ambulance and better good roads (9%).

**Table 1.** Women's (and husband's) socioeconomic, demographic end obstetric history associated with institutional delivery

	Place of delivery					
Characteristics	Hospital		Home		OR (95% CI)	
	No.	%	No.	%	Unadjusted	Adjusted
Age (year)						
<19	3	6.5	13	12.5	1.00	
20-24	29	63.0	47	45.2	2.67 (0.70-10.19)	
25+	14	30.4	44	42.3	1.38 (0.34-5.55)	
Ethnicity of women						
Brahmin-Chhetri	18	39.1	7	6.7	1.00	
Tamang	9	19.6	80	76.9	0.04 (0.01-0.13)	
Other	19	41.3	17	16.3	0.44 (0.15-1.29)	
Occupation of women						
Farmer	22	47.8	85	81.7	1.00	
Housewife	21	45.7	17	16.3	4.77 (2.16-10.54)	
Other*	3	6.5	2	1.9	5.80 (0.91-36.84)	
Education of women						
Illiterate	9	19.6	64	61.5	1.00	1.00
Primary	9	19.6	28	26.9	2.29 (0.82-6.37)	1.21 (0.35-4.27)
Secondary and above	28	60.9	12	11.5	16.59 (6.27-43.80)	5.61 (1.53-20.54)
Occupation of husbands						
Farmer	12	26.1	61	58.7	1.00	
Other jobs**	26	56.5	34	32.7	3.89 (1.74-8.67)	
Formal sector work or work abroad	8	17.4	9	8.7	4.52 (1.45-14.07)	
Education of husbands						
Illiterate	4	8.7	17	16.3	1.00	
Primary	8	17.4	47	45.2	0.72 (0.19-2.71)	
Secondary and above	34	73.9	40	38.5	3.61 (1.11-11.77)	
Number of family members						
3-4	10	21.7	24	23.1	1.00	
5-8	26	56.5	62	59.6	1.01 (0.42-2.40)	
9+	10	21.7	18	17.3	1.33 (0.46-3.88)	
Number of children						
1	27	58.7	44	42.3	3.92 (1.36-11.31)	
2	14	30.4	28	26.9	3.20 (1.02-10.01)	
3 or more	5	10.9	32	30.8	1.00	
Had antenatal check-up						
No	1	2.2	32	30.8	1.00	
Yes	45	97.8	72	69.2	20.0 (2.64-151.51)	
Adverse pregnancy outcome						
No	38	82.6	99	95.2	1.00	1.00
Yes†	8	17.4	5	4.8	4.2 (1.3-13.54)	9.8 (2.31-41.11)
Transportation						
Yes	5	10.9	30	28.8	1.00	
No	41	89.1	74	71.2	1.32 (1.20-9.22)	

*includes teachers, business owners, and tailors;

**includes teachers, business owners, carpenters, labourers, and drivers;

† Any one of the following: miscarriage, abortion, or stillbirth

**Table 2.** Perceived problems by women with access to delivery care (n=150)

	Hospital		Home		p value
Factor	No	%	No	%	
Health facility far away	14	30.4	44	42.3	0.232
No skilled female health worker	9	19.6	12	11.5	0.293
Woman and family members not aware of delivery care	25	54.3	45	43.3	0.282
No delivery care at local level	3	6.5	4	3.8	0.767
No time due to work load in the home	0	0.0	3	2.9	0.595
No transportation	5	10.9	30	28.8	0.028
No money	10	21.7	15	14.4	0.384

Participants listed up to three issues each

### Health problems during delivery and seeking care

All the women were asked about problems they faced during the last delivery of their babies. Only 24 (16%) women reported that they had problems during delivery of their babies. Prolong labour (n=14) was a major health problem, followed by retained placenta (n=6) and excessive bleeding (n=4). Each of the following problems occurred once in a single different woman: fever, breech presentation, difficulty to deliver baby, and retention of placenta. None of these problems was significantly different between women delivered at home and in an institution.

Twenty women sought help for their health problems. Nine of these women sought help from a doctor, eight from a nurse, and three from a traditional healer. However, only a quarter of women sought help immediately after the problems encountered while seven sought care after 2-6 hours, and eight women sought help after six hours.

Women who did not seek any help for their health problems mentioned following as the main reason: or their family did not perceive it to be a major health problem, the hospital was located far away, and they were unable to travel due to their very weak condition.

## DISCUSSION

### Use of delivery care

Our study found that delivery at home was a still common practice, and the use of skilled attendance at birth was still low in the rural area which is close to Kathmandu, the capital of Nepal. However, the percentage of institutional deliveries at the study area was higher than the national average (1). Not a single delivery at home in our study was attended by a skilled attendant. The use of skilled attendants at delivery is the key indicator to reflect towards the Millennium Development Goal (MDG) of improving maternal health (16). The use of professional attendants at delivery at the basic emergency obstetric care facility and in the community has reduced the maternal mortality ratio to 50 or less per 100,000 livebirths (2,3). The shortage of skilled health persons, especially in rural areas, is a major problem in Nepal. The MCHWs could play a key role at the community level as skilled attendants in Nepal (7). No delivery was found to be attended by the MCHWs in this study. The low use of MCHWs might be due to the fact that the local people were not trusting the quality of their services or not having the resources to engage them in delivery. The literature suggests that the quality of care is also an important factor in the use of skilled attendants (16). A few women used TBAs at delivery in our study but TBAs are explicitly excluded from the category of skilled attendant by the WHO (17).

### Factors associated with delivery care

Significantly more women in the VDC D delivered at the hospital. It might be due to the availability of local hospital services, and travel distance to hospitals in Kathmandu, along with other factors, such as use of antenatal care (ANC), age, education, and occupation of women, occupation and education of their husbands, ethnicity, number of children, and adverse obstetric history associated with the use of institutional delivery care.

Attendance of ANC was found to be the best opportunity to motivate women to use skilled delivery care as in other studies (5,12,18). However, 69.2% of women who used ANC delivered babies at home. Results of studies showed that the use of ANC highly correlated with women's health to seek skilled delivery care (16,19). Results of a study in Nepal showed that ethnicity was not significantly associated with place of delivery (19). However, ethnicity was found to be associated with the use of institutional delivery care in our study. Women from Brahmin/Chhetri ethnicity were more likely to deliver babies in hospital. Older women prefer delivery at home and are least likely to use skilled delivery care (16,18,20).

As in our study, occupation of women was often significantly associated with the uptake of skilled delivery care as were occupation and education of their husbands (12,18,20). Education of women was a positive predictor for delivery at hospital in our study. Low maternal education is one of the reasons for delivery at home in Nepal (5,18-23).

Women with three or more children were more likely to deliver baby at home. High parity increases the risk of giving birth outside the health facility by unskilled birth attendants (18,19-22). Likewise, having a history of previous adverse pregnancy outcome was an influential factor to seeking skilled deliver care. In contrast, obstetric history was significantly associated in another study in Nepal (19). The size of the family was not associated with the use of skilled delivery care (19).

### Barriers to access delivery care

The findings of our study revealed many problems to access skilled care at birth. Health facility located far away and lack of transportation facility are the real problems for institutional delivery care in Nepal (18,19,23). Lack of skilled health professional is another main barrier to access skilled care at birth in Nepal (4,5). Not a single delivery at home was assisted by a skilled health worker in our study, which reflects the shortage of reliable skilled attendants in the community (4,5,16). Similarly, due to lack of awareness of delivery care, women and their families in Nepal usually do not seek skilled delivery care unless they encounter complications (14). On the other hand, our study illustrated that 52% of the women decided to deliver their babies at home. It means that women in Nepal prefer delivery at home because it is easy and convenient (24).

Lack of knowledge about pregnancy-related risk factors can be a reason of not perceiving the need of a skilled attendant at delivery (25). Higher cost of delivery at hospital is another major factor causing women to deliver their babies at home (5,16,20). Delivery care provided by skilled attendants is more expensive, which is not affordable for poor women and families in Nepal (21,23,26). Refusal of family to access skilled delivery care may relate to the cost of a delivery. Low socioeconomic status of women also restricts to access skilled delivery care (20,27) and increases the chances of unassisted delivery at home (24).

Results of a participatory intervention study in Nepal showed that women in an intervention cluster were more likely to have hygienic care, such as HDK, during delivery at home (28). Clean and safe birth at home is emphasized in the national maternity care guidelines in Nepal (Ministry of Health, 1996). However, the use of HDK is very low (13,24). Similarly, the use of unclean instruments for cutting the baby's cord is still very common in rural Nepal (14). Such an undesirable practice may cause neonatal tetanus and other serious infections to the baby. Neonatal tetanus is a leading cause of death of newborn babies (29).

### Health problems during delivery and seeking care

Critical health problems, such as prolong labour, retained placenta, and excessive bleeding, during delivery were reported by some women in our study (14). Although most women who had health problems sought professional help, still some (15%) of them sought care from a traditional healer. Women in Nepal are more likely to seek help from traditional healers (30). Such kind of practice may delay women from accessing more appropriate care. The timing for seeking care was quite late in this study. The major obstacles to seeking care are: not perceiving illness (31), a limited capacity to recognize danger-signs; the need to watch and wait; and a great preference to treat illness within the community (14,32); and distance/transportation problem (19,23,25).

### Limitations

During the time of our study, the political situation of Nepal was unstable due to the clash between the Government and Maoist rebellions. The security situation was not safe enough to visit many remote areas, and because of the insurgency, the local people were hesitant to talk to strangers. Data were collected just before the great democratic movement in Nepal. Therefore, the study was limited by the areas which where accessible and relatively safe. Additionally, there were both time and funding restrictions. Moreover, probability sampling, such as simple random-sampling method, could not be applied as planned due to a lack of accurate information about a sampling frame. Therefore, complete enumeration of eligible women was used based on the sampling list provided by the FCHVs.

### Conclusions

Skilled delivery care at birth in Nepal is still very poor. Deliveries at home by unskilled birth attendants are still common, even in a rural area relatively close to the capital city of Nepal. Key factors associated with the uptake of skilled delivery care included: age, ethnicity, occupation, and education of women; occupation and education of their husbands; and number of pregnancies and children, use of ANC, and the experience of problems during pregnancy. The main barriers to accessing skilled delivery were distance to hospital and costs associated with a delivery at hospital. Health problems during delivery, such as long labour and retained placenta, were not common but they were included in the main reasons for seeking intrapartum care late.

### Recommendations

Awareness of delivery care plays a vital role in perceiving the need for skilled delivery care. Therefore, an intervention consisting of awareness programmes promoting delivery care should be implemented targeting women, family, mothers-in-law, and husbands. Moreover, the results of our study suggest that interventions should focus on the Tamang ethnicity, illiterate and farmer women, women with poor obstetric history, and women having more children. More skilled health workers should be made available in the villages. Hygienic technique, such as HDK, should be freely available to all women who would like to have delivery at home. A reliable ambulance service should also be made available to accessible areas while at the same time general improvements of the roads would help reduce transport time.

## ACKNOWLEDGEMENTS

The authors thank all the respondents, the FCHVs, and the VDC chairs who supported the study. They acknowledge the support of many organizations, such as the International Nepal Fellowship, Tuberculosis Leprosy Project, German Institute for Medical Mission, Stichting Supplementiefonds Sonnevanck (The Netherlands), Swiss Friends for Mission to Nepal, Green Tara Trust, UK, and the University of Aberdeen. The authors also thank the editor of *Journal of Health, Population and Nutrition* and the anonymous reviewers for their helpful comments on the original submission.
